# Annexin A3 Knockdown Suppresses Lung Adenocarcinoma

**DOI:** 10.1155/2016/4131403

**Published:** 2016-11-22

**Authors:** Ying-Fu Liu, Qing-Qing Liu, Yue-Hua Zhang, Jing-Hua Qiu

**Affiliations:** ^1^Department of Basic Medical Sciences, Medical College, Xiamen University, Xiamen, Fujian, China; ^2^Laboratory Animal Center, Xiamen University, Xiamen, Fujian, China

## Abstract

Our previous study identified an elevated abundance of annexin A3 (Anxa3) as a novel prognostic biomarker of lung adenocarcinoma (LADC) through quantitative proteomics analysis. However, the biological functions of Anxa3 in LADC are not fully clear. In this study,* in vitro* and* in vivo* assays were performed to investigate the effects of Anxa3 downregulation on the growth, migration, invasion, metastasis, and signaling pathway activation of LADC cells. After Anxa3 downregulation, the growth of A549 and LTEP-a2 LADC cells was slowed and they showed decreased migration and invasion* in vitro*. Anxa3 knockdown significantly inhibited tumor formation by A549 cells* in vivo*; while many metastases were formed by control A549 cells, there were obvious reductions in the numbers of lung, liver, and brain metastases formed by Anxa3 knockdown in A549 cells. Furthermore, Anxa3 knockdown significantly decreased MMP-2 and N-cadherin expression and increased E-cadherin expression both in cell lines* in vitro* and in tumor nodules examined during* in vivo* tumorigenesis assays. Interestingly, Anxa3 downregulation reduced the phosphorylated levels of MEK and ERK. In summary, Anxa3 knockdown inhibited the growth, migration, invasion, and metastasis of LADC, decreased the activation of the MEK/ERK signaling pathway, and modulated the expression of MMP-2, E-cadherin, and N-cadherin.

## 1. Introduction

Lung cancer, with its rapidly growing morbidity, is the leading cause of cancer deaths worldwide, resulting in over 1.3 million deaths per year [1]. In particular, lung adenocarcinoma (LADC), the most common subtype of non-small-cell lung cancer, accounts for 40% of lung cancer incidences [2]. Despite advancements in molecular diagnosis and targeted therapies, the average 5-year survival rate for LADC is approximately 15%, mainly because of cancer cell metastasis and the lack of effective late-stage treatment [3]. Thus, it is urgent to gain a better understanding of the molecular mechanism that regulates the carcinogenesis and metastasis of LADC.

In our previous study, annexin A3 (Anxa3) was identified as a novel metastasis-related protein in LADC using quantitative proteomics, and its high expression was found to be correlated with lymph node metastasis, advanced tumor stage, recurrence, and poor prognosis [4, 5]. However, the biological roles of Anxa3 in the carcinogenesis and progression of LADC are incompletely understood and need to be studied further.

Anxa3 belongs to the annexin family, which is comprised of highly abundant intracellular proteins with calcium-dependent phospholipid binding activities [6]. Recent studies have shown that Anxa3 might function as either a tumor promoter or suppressor in different cancers [7, 8]. As found, the upregulation of Anxa3 expression could promote the development of colorectal cancer and gastric cancer [9–11] and enhance the metastatic activity of LADC [12]. However, in prostatic cancer and renal cancer, it was shown that there is a relationship between Anxa3 downregulation and tumor cells development [13]. It was reported that the expression of Anxa3 was positively correlated with Ki-67 and Bcl-2 expression in gastric cancer [11]. In hepatocellular carcinoma, Anxa3-mediated maintenance of cancer stem-like cells activity was revealed to most likely involve the HIF-1*α*/Notch pathway [14]. Multiple lines of evidence have indicated that Anxa3 expression might be a potential prognostic marker for tumor patients and an indicator of tumor development, invasion, and metastasis.

Our previous study elucidated the relationship between Anxa3 expression and clinicopathological factors of LADC; however, its biological roles in LADC are still not fully clear. In this study, LADC cell lines with stable knockdown of Anxa3 expression were established and their behaviors were investigated* in vitro*. Then, the abilities of the Anxa3 knockdown cells to undergo tumor formation and metastasis* in vivo* were evaluated in nude mice. Furthermore, the effects of Anxa3 downregulation on the activation of key signaling pathways and the expression of some LADC-associated molecules were also investigated. Altogether, the biological functions of Anxa3 in LADC were comprehensively explored in this study.

## 2. Materials and Methods

### 2.1. Cell Culture and Transfection of shRNA Vector

The human LADC cell lines A549 and LTEP-a2 were obtained from the Chinese Academy of Medical Sciences (Shanghai, China). Cells were grown according to standard condition. Anxa3 knockdown vector (shAnxa3) and control vector were constructed by GeneChem (Shanghai, China). The target sequence within the Anxa3 gene was 5′-AAG AGA TTA TCC AGA CTT T-3′, which was queried using the RNAi Consortium's Library Database (http://www.broadinstitute.org/rnai/public/gene/search). The small hairpin RNA (shRNA) sequence targeting Anxa3 was 5′-GTA AGA GAT TAT CCA GAC TTT CTC GAG AAA GTC TGG ATA ATC TCT TAC-3′. The shRNA sequence was cloned into the GV102 vector with enhanced green fluorescent protein and the identity of the resulting construct was verified by sequencing. A549 and LTEP-a2 cells were transfected with shAnxa3 or control vector using Lipofectamine® 2000 transfection reagent (Life Technologies) and G418 was added to select the transfected cells as described previously [15]. The mRNA and protein levels of Anxa3 in all stably transfected cell lines were detected by quantitative PCR (qPCR) and western blotting, respectively.

### 2.2. MTT Assay

MTT assay was applied to detect cell viability. Briefly, cells were seeded onto 96-well culture plates as 2000 cells per well. After incubation, 20 *μ*L MTT (5 mg/mL; Sigma-Aldrich, St. Louis, MO, USA) was added to the medium and the tumor cells were cultured for another 4 h at 37°C. The medium was carefully discarded and then 150 *μ*L dimethyl sulfoxide (Sigma-Aldrich) was added to each well followed by mixing by shaking for 10 min for dissolving the formazan crystal. Next, measuring the absorbance of each well at 490 nm wavelength was to draw the curve of growth.

### 2.3. Colony Formation Assay

Colony formation assay was performed as described in our previous study [15]. In brief, cells were seeded at a density of 500 cells per well in 6-well culture plates and cultured for about two weeks. Then, the medium was discarded and cells were washed with phosphate-buffered saline (PBS) twice. Then, cells were fixed in methanol and stained with crystal violet. Colonies which have more than 50 cells were counted under the microscope. And then the percentage of the number of colonies compared to the control group was calculated.

### 2.4. Wound Healing Assay

For the scratch wound healing assay, subconfluent cells of each group were scraped using sterilized 10 *μ*L pipette tips, washed with PBS, and cultured in normal medium. Wound healing was observed under a CKX41 inverted microscope (Olympus Corporation, Tokyo, Japan) and images were captured daily.

### 2.5. Invasion Assay

The invasion assays were performed using Transwell® chambers (Corning Inc., Corning, NY, USA) with Matrigel® (BD Biosciences, Franklin Lakes, NJ, USA) coated filters as described previously by us [16]. The cells were added to inserts with Matrigel coated filters. DMEM containing 10% (v/v) FBS was placed in the lower chamber as the chemoattractant. After incubation, noninvading cells on the upper side of the membrane were removed with cotton swabs and the invading cells were fixed and stained with 0.1% (w/v) crystal violet.

### 2.6. Western Blot

Briefly, 30 *μ*g protein lysates of each sample were separated by sodium dodecyl sulfate-polyacrylamide gel electrophoresis on a 10% (v/v) polyacrylamide gel and transferred to polyvinylidene fluoride membranes. Membranes were incubated with the primary antibodies overnight at 4°C as follows: anti-Anxa3 (dilution, 1 : 800; Abnova, Taipei, Taiwan), anti-Anxa1 (1 : 1000; Santa Cruz Biotechnology, Dallas, TX, USA), anti-Anxa2 (1 : 1000; R&D Systems, Minneapolis, MN, USA), anti-MEK1/2 (1 : 1000; Cell Signaling Technology, Danvers, MA, USA), anti-phospho-MEK1/2 (1 : 1000; Cell Signaling Technology), anti-ERK1/2 (1 : 1000; Cell Signaling Technology), anti-phospho-ERK1/2 (1 : 1000; R&D Systems), anti-p38 MAPK (1 : 1000; Cell Signaling Technology), anti-phospho-p38 MAPK (1 : 1000; Cell Signaling Technology), anti-Akt (1 : 1000; Cell Signaling Technology), anti-phospho-Akt (1 : 1000; Cell Signaling Technology), anti-I*κ*B*α* (1 : 1000; Cell Signaling Technology), anti-phospho-I*κ*B*α* (1 : 1000; Cell Signaling Technology), anti-matrix metalloproteinase-2 (MMP-2) (1 : 2000; Cell Signaling Technology), anti-E-cadherin (1 : 800; Santa Cruz Biotechnology), anti-N-cadherin (1 : 800; Santa Cruz Biotechnology), anti-GAPDH (1 : 5000; R&D Systems), and anti-*β*-actin (1 : 5000; R&D Systems); and they were incubated with anti-rabbit or mouse horseradish peroxidase-conjugated secondary antibodies. Then, the blots were developed using enhanced chemiluminescence detection system (GE Healthcare, Little Chalfont, Buckinghamshire, UK) and quantitated by densitometry using a Storm™ optical scanner with ImageQuant™ software (GE Healthcare).

### 2.7. Real-Time qPCR

Total RNA was extracted from cells using the SV Total RNA Isolation System (Promega, Madison, WI, USA) according to the manufacturer's instructions, and then reverse transcription was performed using the GoScript™ Reverse Transcription System (Promega) according to the manufacturer's instructions. Then, qPCR was performed with GoTaq® qPCR Master Mix (Promega) according to the manufacturer's instructions using a ViiA™ 7 detection system (Applied Biosystems, Foster City, CA, USA). Gene-specific primers for human Anxa1 (forward: 5′-GGT GAC CGA TCT GAG GAC-3′, reverse: 5′-CTG GTG GTA AGG ATG GTA TT-3′), Anxa2 (forward: 5′-CCA GAA CCA ACC AGG AGC-3′, reverse: 5′-CTT GCG GAA GTC ACC AGA-3′), Anxa3 (forward: 5′-ATC TCA TGG TGG CCC TAG-3′, reverse: 5′-ATT TGC CTG CTT GTC CTG-3′), and ACTB (forward: 5′-GTC ACC AAC TGG GAC GAC A-3′, reverse: 5′-CAC AGC CTG GAT AGC AAC G-3′) were designed using Primer 5.0 software (Premier Biosoft International, Palo Alto, CA, USA) and synthesized by Sangon Biotech Corporation (Shanghai, China). Each PCR reaction was performed in triplicate.

### 2.8. Tumor Formation Assay* In Vivo*

Twenty male BALB/c nude mice aged about 6 weeks were obtained from the Chinese Academy of Sciences (Shanghai, China) and fostered under specific conditions free of pathogen. The nude mice were randomly divided into 2 groups (*n* = 10 per group), and the 2 groups were treated with A549/control and A549/shAnxa3 cells, respectively. Subcutaneous injections of A549/control or A549/shAnxa3 cells (1 × 10^7^ cells/100 *μ*L PBS) were performed in the right flank of the nude mice. Through measuring the length (*L*) and width (*W*) every 2 days, the volume of tumor nodule was calculated according to *L* × *W* ^2^ × 0.5. After about 6 weeks, all mice were killed and the tumors were isolated, weighed, and fixed in formalin for further study. Embedded tissue was sectioned into 4 *μ*m sections and analyzed by immunohistochemistry.

### 2.9. Immunohistochemistry

The tumor tissue sections were analyzed using standard immunohistochemical techniques. The immunohistochemical staining technique was performed as in our previous study [15]. Briefly, tumor tissue sections were firstly incubated with the primary antibody overnight at 4°C and then incubated with the secondary antibody using a MaxVision™ kit (Maixin Co., Fuzhou, China). The immunohistochemical staining was developed using a 3,3′-diaminobenzidine reagent (Maixin Co.). PBS buffer was used for replacing the primary antibodies in negative controls.

### 2.10. Experimental Metastasis Model* In Vivo*

Twenty male BALB/c mice aged 5 weeks obtained from the Chinese Academy of Sciences (Shanghai, China) were fostered as mentioned above. The mice were randomly divided into 2 groups (*n* = 10 per group), which were treated with A549/control and A549/shAnxa3 cells, respectively. 2 × 10^6^ cells were injected into the lateral tail vein of each mouse. The body weights of the mice were measured every 2 days. About 45 days later, all the mice were killed and organs including lung, liver, and brain were removed and examined for metastatic tumor formation. The number of tumor nodules was counted and organs were fixed in 10% (w/v) formalin and then embedded in paraffin for further study.

### 2.11. Statistical Analysis

Software SPSS 10.0 (IBM, Armonk, NY, USA) was applied for all statistical analyses in this study. All experiments were repeated 3 times. Differences of *p* < 0.05 were to be considered as significant.

## 3. Results

### 3.1. Anxa3 Knockdown Inhibited LADC Growth* In Vitro*

**Figure 1:**
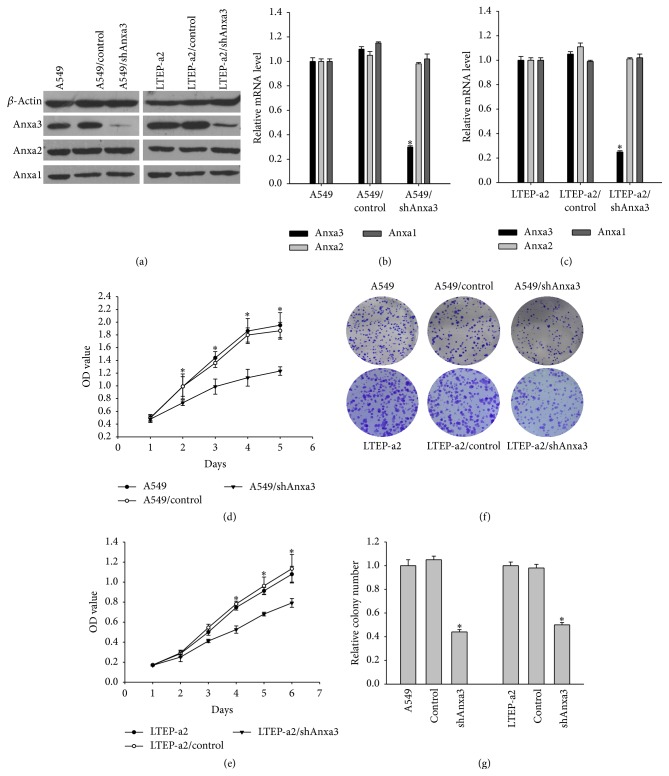
Detection of cell viability after Anxa3 downregulation. (a) Western blot and qPCR (b and c) showed Anxa3, Anxa2, and Anxa1 expression levels in stably transfected shAnxa3 or control shRNA cell lines. MTT assay (d, e) showed that cell viability decreased after Anxa3 downregulation. Colony formation assays (f) and (g) histograms of relative colony numbers indicated that the knockdown of Anxa3 expression significantly reduced colony formation by LADC cells. *∗* indicates *p* < 0.05.

To elucidate the biological roles of Anxa3 in LADC, the stably transfected cell lines A549/shAnxa3, LTEP-a2/shAnxa3, A549/control, and LTEP-a2/control were constructed. As shown in Figures 1(a)–1(c), Anxa3 mRNA and protein levels in A549/shAnxa3 and LTEP-a2/shAnxa3 cells were decreased significantly compared with those of their corresponding control cells, while the mRNA and protein levels of Anxa1 and Anxa2, which belong to the same annexin family as Anxa3, were not significantly different between the shAnxa3 and control cells. Then, the growth ability after downregulating Anxa3 was detected further. The results of MTT assay showed a significant reduction of viable A549/shAnxa3 cells from the second day of culture and LTEP-a2/shAnxa3 cells from the third day, compared with that of their corresponding control and null cells on each day, respectively (Figures 1(d) and 1(e)). Colony formation assay analysis showed fewer colonies in A549/shAnxa3 and LTEP-a2/shAnxa3 cells than in their corresponding control and null cells (Figures 1(f) and 1(g)). These results indicated that knockdown of Anxa3 expression could significantly inhibit the growth of LADC cells* in vitro*.

### 3.2. Anxa3 Knockdown Significantly Suppressed the Migration and Invasion of LADC Cells

**Figure 2:**
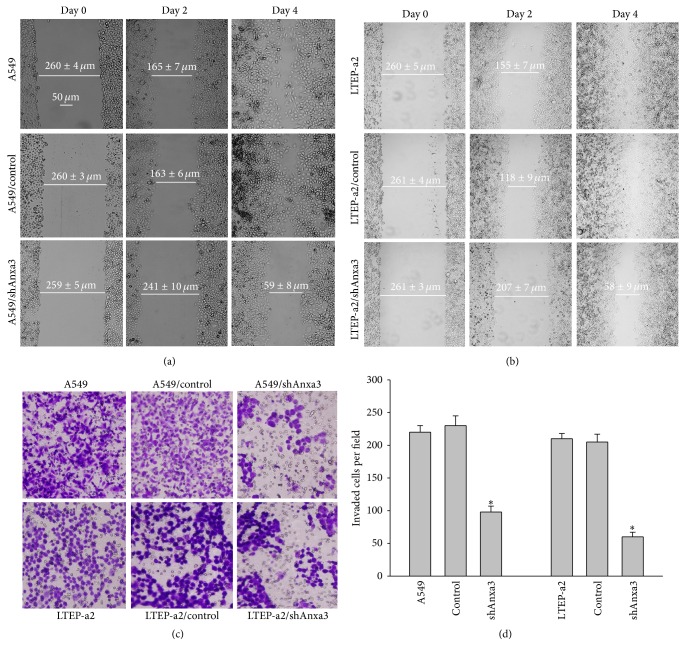
Wound healing assays indicated that downregulating Anxa3 expression inhibited the migration of A549 (a) and LTEP-a2 (b). Magnification: ×100. Transwell assay (c) and analysis of histograms (d) of the numbers of invaded cells indicated that downregulating Anxa3 expression significantly decreased the invasive behavior of LADC cells. Magnification: ×200. *∗* indicates *p* < 0.05.

To investigate the effects of Anxa3 downregulation on the migration and invasion of LADC* in vitro*, wound healing and invasion assays were performed. As shown in Figure 2(a), by the fourth day after performing scratch wounding, the scratch wounds of A549/control and A549/null cells had almost disappeared, while that of A549/shAnxa3 cells was not healed. The scratch wound healing of LTEP-a2 cells showed the same change with Anxa3 knockdown as that of A549 cells (Figure 2(b)), indicating that knockdown of Anxa3 expression consistently and significantly suppressed the migration of LADC cells. The invasion assay results (Figures 2(c) and 2(d)) showed that the numbers of cells that invaded into the A549/shAnxa3 and LTEP-a2/shAnxa3 groups were obviously lower than those in their corresponding control and null cell groups. These results showed that the invasive ability of LADC cells was significantly reduced by Anxa3 downregulation.

### 3.3. Anxa3 Knockdown Altered the Expression of Effector Molecules in LADC Cells

**Figure 3:**
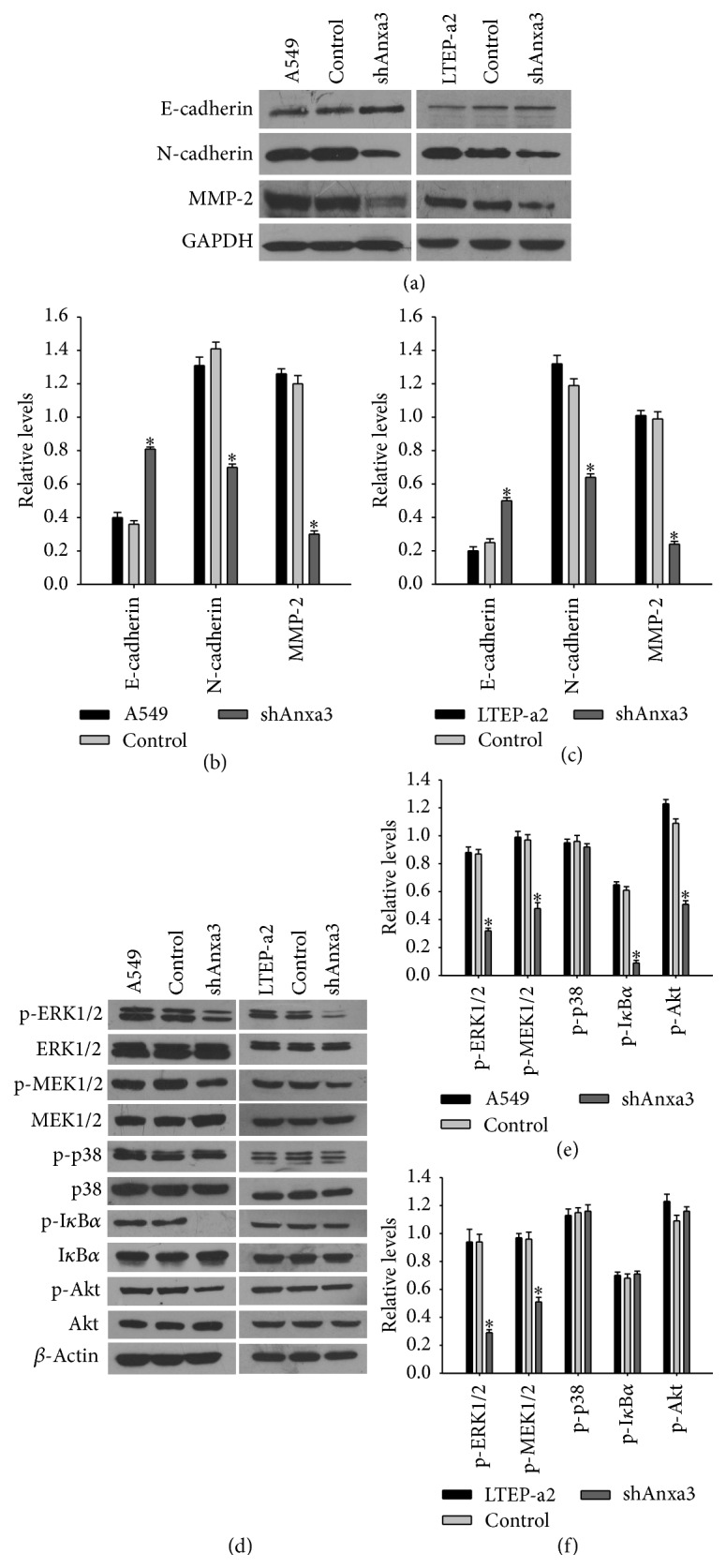
Knockdown of Anxa3 expression affected the expression of some effector molecules and some signaling molecules. (a) Western blot analysis of the expression of MMP-2, N-cadherin, and E-cadherin. (b, c) Histograms of the relative expressions of the effector molecules. *∗* indicates *p* < 0.05. (d) Western blot analysis of the expression of some cancer-associated signaling molecules in A549 and LTEP-a2 cells and their corresponding stably transfected cell lines. (e, f) Histograms of the relative expressions of the signaling molecules. *∗* indicates *p* < 0.05.

The results described above showed that the downregulation of Anxa3 expression significantly inhibited the migration and invasion of LADC cells. MMPs, E-cadherin, and N-cadherin are well-known regulators of the migration and invasion of cancer cells, and MMP-2 is the most highly expressed MMP in lung cancer. Therefore, we investigated the expression of MMP-2, E-cadherin, and N-cadherin following Anxa3 knockdown in LADC cells. As shown in Figures 3(a)–3(c), compared with the control and null cells, shAnxa3 cells had significantly lower MMP-2 and N-cadherin expression and significantly higher E-cadherin expression. The results indicated that downregulating Anxa3 expression reduced MMP-2 and N-cadherin expression but enhanced E-cadherin expression in LADC cells.

### 3.4. Anxa3 Knockdown Decreased MEK/ERK Pathway Activation in LADC Cells

To explore the biological roles of Anxa3 in LADC, we analyzed the changes in the activation of several important cancer-associated signaling pathways induced by Anxa3 downregulation. As revealed in Figures 3(d)–3(f), in A549 cells, the phosphorylation of MEK1/2, ERK1/2, Akt, and I*κ*B*α* was significantly reduced after Anxa3 downregulation, while there was no change in the phosphorylation of p38 MAPK. However, in LTEP-a2 cells, only the phosphorylation levels of MEK1/2 and ERK1/2 were decreased significantly by Anxa3 knockdown, while those of Akt, I*κ*B*α*, and p38 MAPK were not altered. These results indicated that downregulating Anxa3 expression inactivated the MEK/ERK pathway in the A549 and LTEP-a2 LADC cell lines, suggesting that the MEK/ERK pathway is a target of Anxa3 in LADC.

### 3.5. Anxa3 Knockdown Inhibited LADC Tumor Formation* In Vivo*

**Figure 4:**
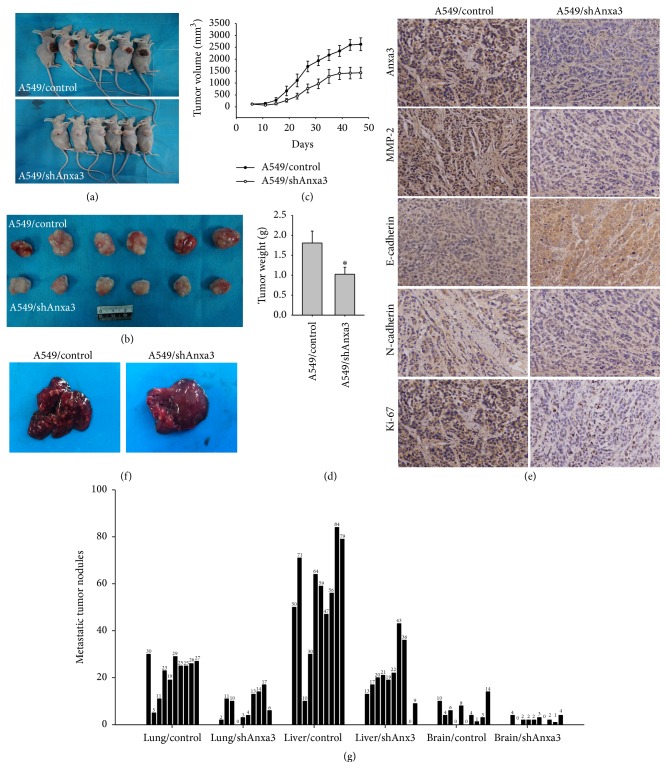
Downregulating Anxa3 expression inhibited tumor growth and tumor metastasis* in vivo*. Representative pictures of nude mice (a), tumor nodules (b), the curve of tumor growth (c), and the mean weights (d) of tumor nodules in A549/control and A549/shAnxa3 group were shown. *∗* indicates *p* < 0.05. Immunohistochemical analyses (e) of Anxa3, MMP-2, E-cadherin, N-cadherin, and Ki-67 expression in tissue sections of tumor nodules in the A549/control and A549/shAnxa3 groups were shown. Magnification: ×200. Representative pictures of metastatic tumor nodules in the livers (f) and histograms of metastatic tumor nodules (g) of lung, liver, and brain in the A549/control and A549/shAnxa3 groups were shown. *∗* indicates *p* < 0.05.

The assays* in vitro* as described above showed that knockdown of Anxa3 expression could inhibit the growth of LADC cells. Next, we further elucidated the effect on tumor formation* in vivo* by downregulating Anxa3 expression. As result, the curve of tumor growth (Figure 4(c)) showed that the tumor volumes of the A549/shAnxa3 group were significantly decreased compared with those of the A549/control group from about 25 days after injection. And the tumor nodules isolated from the A549/control group were significantly larger than those of the A549/shAnxa3 group (Figure 4(b)). Moreover, most of the tumor nodules in A549/control group showed necrosis that was evident as brown coloration on the surface of the nodules (Figure 4(a)). Statistical analysis found that the mean tumor volume of the A549/shAnxa3 group was significantly smaller than that of the A549/control group, as was the mean tumor weight (Figure 4(d)). All of these results demonstrated that knocking down Anxa3 expression inhibited tumor formation* in vivo*. Then, we made the tumor nodules into sections to validate MMP-2, E-cadherin, and N-cadherin expression alteration. Immunohistochemical results found that almost no Anxa3 expression was detected in the tumor nodules of the shAnxa3 group, which verified that the knockdown of Anxa3 was successfully maintained* in vivo*. In addition, the expression of MMP-2 and N-cadherin was reduced while that of E-cadherin was increased in A549/shAnxa3 tumors (Figure 4(e)), similar to the results shown in cell lines* in vitro*. In addition, we analyzed a proliferation marker, Ki-67 expression in the tumor nodules. It was found that Ki-67 expression was significantly lower in shAnxa3 tumors than in control tumors, which further verified that Anxa3 downregulation could inhibit the growth of LADC.

### 3.6. Anxa3 Knockdown Significantly Suppressed LADC Metastasis* In Vivo*

A representative picture of metastatic tumor nodules in liver from the control and shAnxa3 groups is shown in Figure 4(f). The number of metastatic tumor nodules in liver of the shAnxa3 group was significantly less than that of the control group. Histogram analysis of metastatic tumor nodules in lung, liver, and brain from the control and shAnxa3 groups was shown in Figure 4(g). Compared to the control group, the numbers of metastatic tumor nodules in the lung, liver, and brain of the shAnxa3 group were all significantly lower. These results indicated that Anxa3 knockdown effectively suppressed LADC cells metastasis* in vivo*.

## 4. Discussion

In this study, in the A549 and LTEP-a2 LADC cell lines, viable cells and colony formation were significantly decreased after Anxa3 downregulation. Furthermore, investigation by* in vivo* tumorigenesis assay also showed that tumor formation by injected LACD cells in nude mice of the A549/shAnxa3 group was inferior to that in mice of the A549/control group. These results indicated that Anxa3 knockdown powerfully inhibited the growth of LADC* in vitro *and* in vivo*. Furthermore, the tumors of nude mice of the shAnxa3 group showed reduced Ki-67 expression compared with that in mice of the control group, suggesting that Anxa3 affects cell proliferation in LADC. Moreover, the expression of Anxa3 was reported to be positively correlated with Ki-67 expression in gastric cancer [11]. All of the above results indicated that Anxa3 plays a vital role in cancer cell growth.

In addition, this study found that the migration and invasion of shAnxa3 cells were significantly decreased compared with those of control or null cells. Experiments using an* in vivo* model of metastasis showed that the numbers of metastatic tumor nodules in organs including the liver, lung, and brain of the shAnxa3 group were significantly decreased compared to those in the control group. The above results indicated that Anxa3 downregulation could suppress the migration, invasion, and metastasis of LADC cells. Similarly, Yu et al. reported that silencing endogenous Anxa3 suppressed the proliferation, migration, and invasion of gastric cancer cells [17]. As we know, MMP-2, E-cadherin, and N-cadherin are effector molecules with key roles in the migration and invasion of tumors [18–20]. Therefore, the effects of Anxa3 downregulation on the expression of these molecules warranted further investigation. Interestingly, in the cell lines* in vitro* and tumor nodules from nude mice* in vivo*, downregulating Anxa3 expression significantly decreased MMP-2 and N-cadherin expression and increased E-cadherin expression. In combination, the above results suggest that downregulating Anxa3 expression may suppress the migration, invasion, and metastasis of LADC through decreasing MMP-2 and N-cadherin expression and increasing E-cadherin expression.

We also investigated the potential regulation of cancer-associated signaling pathways by Anxa3 through screening for the altered expression of some common signaling molecules after Anxa3 downregulation. Decreased phosphorylation of MEK1/2, ERK1/2, Akt, and I*κ*B*α* was detected after downregulating Anxa3 expression in A549 cells. However, only the phosphorylation levels of MEK1/2 and ERK1/2 were decreased after Anxa3 downregulation, and the phosphorylation of Akt, I*κ*B*α*, and p38 MAPK was not altered in LTEP-a2 cells. These results indicated that downregulating Anxa3 expression effectively reduced the activation of the MEK/ERK pathway in LADC cells, which plays important roles in cell growth, invasion, and survival in various types of cancer [21]. However, the reasons why Akt, I*κ*B*α*, and p38 MAPK showed different changes between A549 cells and LTEP-a2 cells may be further studied. From all of the results, we speculated that knocking down Anxa3 expression could suppress the growth, migration, invasion, and metastasis of LADC and these actions may be partly mediated through inhibiting the activation of MEK/ERK pathway. Further research is required to confirm and elucidate this potential mechanism.

Taken together, this study elucidated the biological functions of Anxa3 in the carcinogenesis and development of LADC and further uncovered its role as a regulator of the expression of certain effector molecules such as MMP-2, E-cadherin, and N-cadherin and the activation of MEK/ERK pathway. The findings indicated that Anxa3 plays key roles in LADC; therefore, it may be a new therapeutic target.
